# Crystal structure of 1,3-dihy­droxy-2-(hy­droxy­meth­yl)propan-2-aminium 2-(4-iso­butyl­phen­yl)propano­ate: a simple organic salt of racemic ibuprofen

**DOI:** 10.1107/S2056989015012979

**Published:** 2015-07-11

**Authors:** Benyong Lou

**Affiliations:** a Department of Chemistry and Chemical Engineering, Minjiang University, Fuzhou 350108, People’s Republic of China

**Keywords:** crystal structure, ibuprofen, trometamol, mol­ecular salt, hydrogen bonding, chains, sheets

## Abstract

In the title organic salt of ibuprofen with trometamol, C_4_H_12_NO_3_^+^·C_13_H_17_O_2_^−^, the carb­oxy­lic acid group of ibuprofen has transferred its proton to the amino N atom of trometamol. In the crystal, the trometamol cations are linked *via* N—H⋯O hydrogen bonds, forming chains along [001]. To these chains are attached the ibuprofen anions *via* O—H⋯O and N—H⋯O hydrogen bonds. The chains are linked *via* further N—H⋯O and O—H⋯O hydrogen bonds, forming sheets parallel to (100). Two C atoms of the propano­ate substituent in the ibuprofen anion are disordered over two sets of sites and were refined with a fixed occupancy ratio of 0.7:0.3.

## Abstract 2

The title compound is an organic salt of ibuprofen with trometamol, in which the carb­oxy­lic acid group of ibuprofen has transferred its proton to the amine N atom of trometamol. In the crystal, the ions are linked by a series of O—H⋯O and N—H⋯O hydrogen bonds, forming sheets parallel to (100).

## Chemical context   

Salt formation is an effective approach for modifying the properties of active pharmaceutical ingredients (APIs) (Childs *et al.*, 2007 ▸). Tris(hy­droxy­meth­yl)amino methane, commonly called trometamol, has been successfully exploited for improving the properties of APIs such as ketoprofen (Zippel & Wagenitz, 2006 ▸). In this study, trometamol was employed to crystallize with ibuprofen, giving rise to a new crystalline form, whose crystal structure is reported on herein.
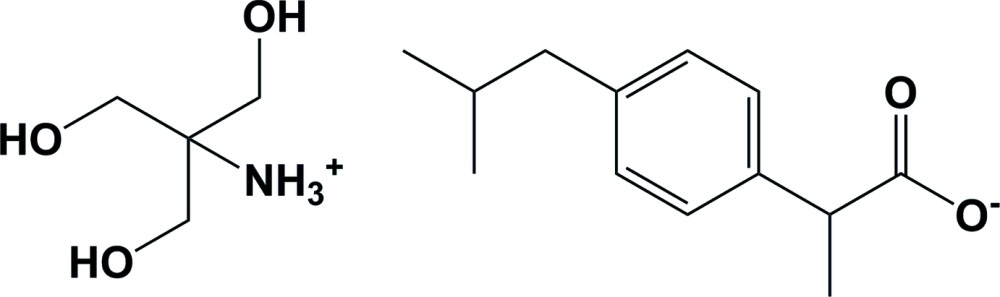


## Structural commentary   

**Figure 1:**
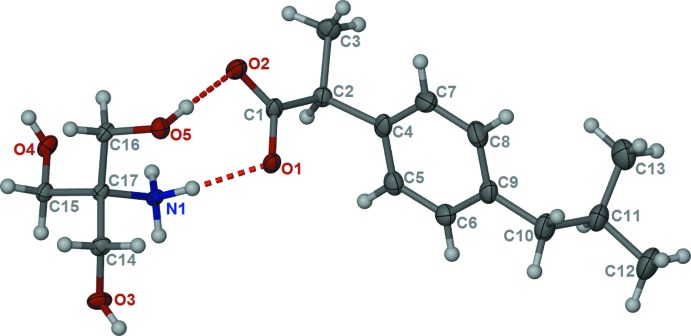
The mol­ecular structure of the title mol­ecular salt, with atom labeling. Displacement ellipsoids are drawn at the 30% probability level. Hydrogen bonds are shown as dashed lines (see Table 1 ▸ for details). The minor components of the disordered atoms (*viz.* C2 and C3) have been omitted for clarity in all three figures.

The mol­ecular structure of the title mol­ecular salt is shown in Fig. 1 ▸. From difference Fourier maps, it was shown that the carb­oxy­lic group of ibuprofen has transferred its proton to the amino N atom of trometamol. This is supported by the C—O bond distances of the carboxyl­ate group of the ibuprofen anion, which are 1.252 (2) and 1.251 (2) Å for C1—O1 and C1—O2, respectively. The carboxyl­ate anion inter­acts with one hydroxyl group of the trometamol cation through a strong hydrogen bond [O5⋯O2 = 2.730 (2) Å; Table 1 ▸]. There also exist hydrogen-bonding inter­actions between the carboxyl­ate anion and aminium H atoms of the cation [N1⋯O1 = 2.763 (2) Å; Table 1 ▸].

## Supra­molecular features   

**Figure 2:**
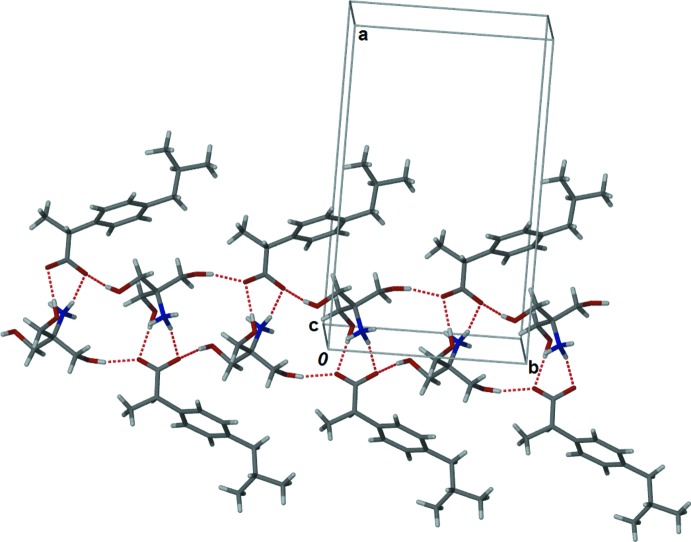
Part of the crystal structure of the title salt, viewed along the *c* axis, showing the hydrogen bonds (dashed lines) forming chains along [001]; see Table 1 ▸ for details.

**Figure 3:**
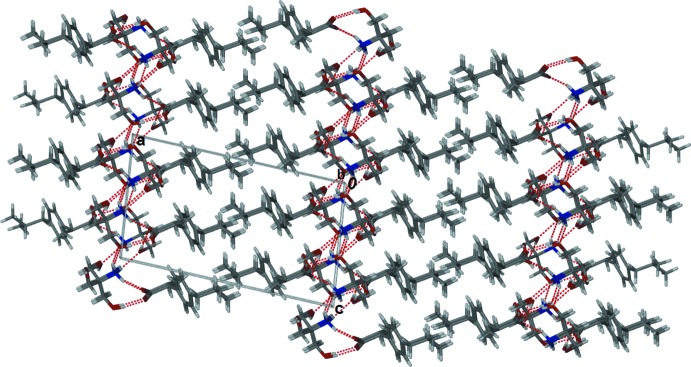
Part of the crystal structure of the title salt, viewed along the *b* axis, showing the sheets parallel to (100) formed by hydrogen bonding (dashed lines; see Table 1 ▸ for details).

In the crystal, the trometamol cations are linked *via* N—H⋯O hydrogen bonds, forming chains along [010]; Table 1 ▸ and Fig. 2 ▸. To these chains are attached the ibuprofen anions *via* N—H⋯O and O—H⋯O hydrogen bonds. The chains are linked *via* further O—H⋯O and N—H⋯O hydrogen bonds, forming sheets parallel to (100); Table 1 ▸ and Fig. 3 ▸.

## Database survey   

A search of the Cambridge Structural Database (Version 5.36, May 2015; Groom & Allen, 2014 ▸) revealed only one hit for organic salts of racemic ibuprofen, *viz.* benzyl­ammonium 2-(4-iso­butyl­phen­yl)propionate 2-(4-iso­butyl­phen­yl)propionic acid (refcode VUCHUX; Molnár *et al.*, 2009 ▸). In fact, it is a salt co-crystal based on ibuprofen and the organic salt (Sun, 2013 ▸). The title compound is the first crystal structure of a simple organic salt of racemic ibuprofen.

## Synthesis and crystallization   

Ibuprofen (206 mg, 1 mmol) and trometamol (121 mg, 1 mmol) were dissolved in methanol (15 mL). The resulting solution was kept in air and after several days colorless plate-like crystals were obtained.

## Refinement   

Crystal data, data collection and structure refinement details are summarized in Table 2 ▸. The hydroxyl and and aminium H atoms were located in difference Fourier maps and freely refined. Two C atoms, C3 and C2, of the propano­ate substit­uent in the ibuprofen anion are disordered over two sets of sites (C3/C3′and C2/C2′) and were refined with a fixed occupancy ratio of 0.7:0.3. H atoms H2 and H2′ were refined with distance restraints C—H = 0.98 (2) Å with *U*_iso_(H) = 1.2*U*_eq_(C). The remainder of the C-bound H atoms were positioned geometrically and refined as riding atoms: C—H = 0.95–1.00 Å with *U*_iso_(H) = 1.5*U*_eq_(C) for methyl H atoms and 1.2*U*_eq_(C) for other H atoms.

## Supplementary Material

Crystal structure: contains datablock(s) I, Global. DOI: 10.1107/S2056989015012979/su5165sup1.cif

Structure factors: contains datablock(s) I. DOI: 10.1107/S2056989015012979/su5165Isup2.hkl

Supporting information file. DOI: 10.1107/S2056989015012979/su5165Isup3.cml

CCDC reference: 1410786

Additional supporting information:  crystallographic information; 3D view; checkCIF report

## Figures and Tables

**Table 1:** Hydrogen-bond geometry (, )

*D*H*A*	*D*H	H*A*	*D* *A*	*D*H*A*
O3H3O2^i^	0.90(3)	1.84(3)	2.725(2)	167(2)
O4H4O1^ii^	0.79(2)	1.92(3)	2.689(2)	163.7(18)
O5H5*A*O1	0.86(2)	2.57(2)	3.0825(19)	119.5(17)
O5H5*A*O2	0.86(2)	1.88(2)	2.730(2)	168.1(18)
N1H1*A*O1	0.94(2)	1.85(2)	2.763(2)	162.9(18)
N1H1*B*O4^iii^	0.94(2)	2.09(2)	2.9224(19)	146.7(16)
N1H1*C*O5^iv^	0.91(2)	1.97(2)	2.806(2)	152.1(18)

Symmetry codes: (i) 
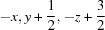
; (ii) 
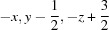
; (iii) 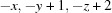; (iv) 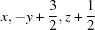.

**Table 2:** Experimental details

Crystal data	
Chemical formula	C_4_H_12_NO_3_^+^C_13_H_17_O_2_
*M*_r_	327.41
Crystal system, space group	Monoclinic, *P*2_1_/*c*
Temperature (K)	173
*a*, *b*, *c* ()	17.523(7), 10.400(4), 9.976(4)
()	97.032(7)
*V* (^3^)	1804.3(12)
*Z*	4
Radiation type	Mo *K*
(mm^1^)	0.09
Crystal size (mm)	0.29 0.22 0.04

Data collection	
Diffractometer	Rigaku Mercury CCD
Absorption correction	Multi-scan (*CrystalClear*; Rigaku, 2000 ▸)
*T*_min_, *T*_max_	0.914, 1.000
No. of measured, independent and observed [*I* > 2(*I*)] reflections	13747, 4096, 3391
*R*_int_	0.028
(sin /)_max_ (^1^)	0.649

Refinement	
*R*[*F* ^2^ > 2(*F* ^2^)], *wR*(*F* ^2^), *S*	0.058, 0.148, 1.11
No. of reflections	4096
No. of parameters	260
No. of restraints	6
H-atom treatment	H atoms treated by a mixture of independent and constrained refinement
_max_, _min_ (e ^3^)	0.22, 0.23

Computer programs: *CrystalClear* (Rigaku, 2000 ▸), *SHELXS97* and *SHELXL97* (Sheldrick, 2008 ▸), *SHELXL2014* (Sheldrick, 2015 ▸) and *X-SEED* (Barbour, 2001 ▸).

## supplementary crystallographic information

### Crystal data

**Table:**

C_4_H_12_NO_3_^+^·C_13_H_17_O_2_^−^	*F*(000) = 712
*M**_r_* = 327.41	*D*_x_ = 1.205 Mg m^−^^3^
Monoclinic, *P*2_1_/*c*	Mo *K*α radiation, λ = 0.71073 Å
*a* = 17.523 (7) Å	Cell parameters from 4551 reflections
*b* = 10.400 (4) Å	θ = 2.8–27.5°
*c* = 9.976 (4) Å	µ = 0.09 mm^−^^1^
β = 97.032 (7)°	*T* = 173 K
*V* = 1804.3 (12) Å^3^	Plate, colorless
*Z* = 4	0.29 × 0.22 × 0.04 mm

### Data collection

**Table:**

Rigaku Mercury CCD diffractometer	4096 independent reflections
Radiation source: fine-focus sealed tube	3391 reflections with *I* > 2σ(*I*)
Graphite monochromator	*R*_int_ = 0.028
Detector resolution: 28.5714 pixels mm^-1^	θ_max_ = 27.5°, θ_min_ = 2.3°
CCD_Profile_fitting scans	*h* = −22→22
Absorption correction: multi-scan (*CrystalClear*; Rigaku, 2000)	*k* = −13→13
*T*_min_ = 0.914, *T*_max_ = 1.000	*l* = −12→12
13747 measured reflections	

### Refinement

**Table:**

Refinement on *F*^2^	6 restraints
Least-squares matrix: full	Hydrogen site location: mixed
*R*[*F*^2^ > 2σ(*F*^2^)] = 0.058	H atoms treated by a mixture of independent and constrained refinement
*wR*(*F*^2^) = 0.148	*w* = 1/[σ^2^(*F*_o_^2^) + (0.0646*P*)^2^ + 0.4122*P*] where *P* = (*F*_o_^2^ + 2*F*_c_^2^)/3
*S* = 1.11	(Δ/σ)_max_ = 0.001
4096 reflections	Δρ_max_ = 0.22 e Å^−^^3^
260 parameters	Δρ_min_ = −0.23 e Å^−^^3^

### Special details

**Table:**

Geometry. All esds (except the esd in the dihedral angle between two l.s. planes) are estimated using the full covariance matrix. The cell esds are taken into account individually in the estimation of esds in distances, angles and torsion angles; correlations between esds in cell parameters are only used when they are defined by crystal symmetry. An approximate (isotropic) treatment of cell esds is used for estimating esds involving l.s. planes.

### Fractional atomic coordinates and isotropic or equivalent isotropic displacement parameters (Å^2^)

**Table:**

	*x*	*y*	*z*	*U*_iso_*/*U*_eq_	Occ. (<1)
O1	0.11773 (7)	0.77640 (11)	0.78429 (13)	0.0439 (3)	
O2	0.13430 (7)	0.58002 (12)	0.71039 (15)	0.0544 (4)	
C1	0.15768 (10)	0.67741 (15)	0.7767 (2)	0.0422 (4)	
C2	0.23375 (17)	0.6782 (3)	0.8754 (4)	0.0408 (6)	0.7
H2	0.2182 (17)	0.670 (3)	0.966 (2)	0.049*	0.7
C3	0.28440 (19)	0.5630 (3)	0.8537 (4)	0.0610 (9)	0.7
H3A	0.2992	0.5660	0.7622	0.091*	0.7
H3B	0.2559	0.4835	0.8652	0.091*	0.7
H3C	0.3307	0.5654	0.9197	0.091*	0.7
C2'	0.2464 (4)	0.6735 (7)	0.8061 (9)	0.0417 (16)	0.3
H2'	0.271 (3)	0.616 (2)	0.746 (6)	0.050*	0.3
C3'	0.2585 (5)	0.5894 (9)	0.9312 (11)	0.070 (2)	0.3
H3'1	0.3115	0.5989	0.9744	0.105*	0.3
H3'2	0.2490	0.4993	0.9055	0.105*	0.3
H3'3	0.2228	0.6156	0.9945	0.105*	0.3
C4	0.27435 (10)	0.80792 (18)	0.8575 (2)	0.0542 (5)	
C5	0.26039 (11)	0.9010 (2)	0.9485 (2)	0.0540 (5)	
H5	0.2281	0.8817	1.0157	0.065*	
C6	0.29208 (11)	1.0220 (2)	0.9450 (2)	0.0548 (5)	
H6	0.2825	1.0830	1.0118	0.066*	
C7	0.32041 (12)	0.8410 (2)	0.7599 (3)	0.0667 (6)	
H7	0.3315	0.7785	0.6955	0.080*	
C8	0.35065 (13)	0.9638 (2)	0.7543 (3)	0.0682 (6)	
H8	0.3813	0.9843	0.6850	0.082*	
C9	0.33732 (11)	1.05711 (19)	0.8470 (2)	0.0549 (5)	
C10	0.37079 (14)	1.1906 (2)	0.8433 (3)	0.0768 (7)	
H10A	0.3344	1.2522	0.8768	0.092*	
H10B	0.3755	1.2131	0.7482	0.092*	
C11	0.44861 (14)	1.2067 (2)	0.9256 (3)	0.0768 (7)	
H11	0.4438	1.1754	1.0190	0.092*	
C12	0.4706 (2)	1.3480 (3)	0.9369 (4)	0.1136 (12)	
H12A	0.4710	1.3842	0.8463	0.170*	
H12B	0.5219	1.3565	0.9878	0.170*	
H12C	0.4331	1.3945	0.9839	0.170*	
C13	0.51137 (16)	1.1303 (3)	0.8743 (4)	0.0991 (10)	
H13A	0.5186	1.1600	0.7834	0.149*	
H13B	0.4973	1.0391	0.8708	0.149*	
H13C	0.5593	1.1419	0.9348	0.149*	
O3	−0.15157 (8)	0.82574 (12)	0.84440 (14)	0.0511 (4)	
H3	−0.1538 (14)	0.910 (2)	0.825 (2)	0.081 (8)*	
O4	−0.08544 (7)	0.44727 (12)	0.91701 (13)	0.0419 (3)	
H4	−0.0903 (12)	0.387 (2)	0.868 (2)	0.066 (7)*	
O5	−0.00842 (7)	0.64538 (11)	0.58848 (12)	0.0397 (3)	
H5A	0.0387 (12)	0.6268 (18)	0.616 (2)	0.059 (6)*	
N1	−0.01557 (8)	0.68283 (13)	0.87045 (14)	0.0313 (3)	
H1A	0.0234 (12)	0.7199 (19)	0.826 (2)	0.049 (5)*	
H1B	0.0080 (11)	0.6126 (19)	0.9193 (19)	0.044 (5)*	
H1C	−0.0290 (11)	0.745 (2)	0.927 (2)	0.046 (5)*	
C14	−0.13080 (10)	0.75903 (15)	0.73082 (18)	0.0411 (4)	
H14A	−0.0997	0.8157	0.6790	0.049*	
H14B	−0.1776	0.7335	0.6710	0.049*	
C15	−0.13077 (9)	0.55121 (15)	0.85901 (17)	0.0367 (4)	
H15A	−0.1507	0.6012	0.9317	0.044*	
H15B	−0.1753	0.5168	0.7991	0.044*	
C16	−0.05760 (10)	0.56874 (15)	0.65824 (17)	0.0370 (4)	
H16A	−0.0300	0.4895	0.6910	0.044*	
H16B	−0.1030	0.5430	0.5950	0.044*	
C17	−0.08440 (9)	0.63973 (14)	0.77797 (16)	0.0310 (3)	

### Atomic displacement parameters (Å^2^)

**Table:**

	*U*^11^	*U*^22^	*U*^33^	*U*^12^	*U*^13^	*U*^23^
O1	0.0357 (6)	0.0295 (6)	0.0647 (8)	−0.0003 (5)	−0.0012 (6)	0.0038 (6)
O2	0.0452 (7)	0.0361 (7)	0.0795 (10)	0.0024 (5)	−0.0018 (7)	−0.0067 (6)
C1	0.0332 (8)	0.0296 (8)	0.0625 (12)	−0.0022 (6)	0.0010 (8)	0.0099 (8)
C2	0.0370 (15)	0.0368 (14)	0.047 (2)	0.0024 (11)	0.0000 (14)	0.0053 (15)
C3	0.0481 (18)	0.0458 (16)	0.084 (3)	0.0132 (13)	−0.0108 (17)	0.0029 (18)
C2'	0.038 (3)	0.042 (3)	0.046 (4)	0.001 (2)	0.007 (3)	0.006 (3)
C3'	0.054 (5)	0.063 (5)	0.087 (7)	0.001 (4)	−0.009 (4)	0.022 (5)
C4	0.0293 (8)	0.0450 (10)	0.0838 (15)	−0.0027 (7)	−0.0115 (9)	0.0023 (10)
C5	0.0395 (10)	0.0578 (12)	0.0636 (13)	−0.0034 (8)	0.0025 (9)	0.0059 (10)
C6	0.0416 (10)	0.0551 (11)	0.0667 (14)	−0.0019 (9)	0.0028 (9)	−0.0108 (10)
C7	0.0480 (12)	0.0656 (13)	0.0859 (17)	−0.0039 (10)	0.0058 (11)	−0.0294 (12)
C8	0.0493 (12)	0.0800 (16)	0.0777 (16)	−0.0129 (11)	0.0180 (11)	−0.0067 (13)
C9	0.0368 (9)	0.0496 (11)	0.0763 (15)	−0.0067 (8)	−0.0007 (9)	−0.0016 (10)
C10	0.0570 (13)	0.0523 (13)	0.119 (2)	−0.0110 (10)	0.0007 (13)	0.0100 (13)
C11	0.0639 (15)	0.0552 (13)	0.112 (2)	−0.0196 (11)	0.0132 (14)	−0.0071 (13)
C12	0.097 (2)	0.0659 (17)	0.177 (4)	−0.0342 (16)	0.015 (2)	−0.016 (2)
C13	0.0588 (16)	0.094 (2)	0.143 (3)	−0.0092 (14)	0.0058 (17)	−0.006 (2)
O3	0.0624 (9)	0.0323 (6)	0.0598 (9)	0.0120 (6)	0.0124 (7)	0.0012 (6)
O4	0.0542 (8)	0.0286 (6)	0.0411 (7)	−0.0050 (5)	−0.0013 (6)	0.0064 (5)
O5	0.0401 (7)	0.0417 (6)	0.0372 (7)	−0.0026 (5)	0.0048 (5)	0.0080 (5)
N1	0.0359 (7)	0.0243 (6)	0.0330 (8)	−0.0003 (5)	0.0014 (6)	−0.0004 (6)
C14	0.0453 (10)	0.0324 (8)	0.0439 (10)	0.0046 (7)	−0.0021 (8)	0.0023 (7)
C15	0.0387 (8)	0.0306 (8)	0.0406 (10)	−0.0046 (6)	0.0043 (7)	0.0025 (7)
C16	0.0453 (9)	0.0310 (8)	0.0344 (9)	−0.0065 (7)	0.0041 (7)	−0.0010 (7)
C17	0.0331 (8)	0.0258 (7)	0.0331 (8)	−0.0017 (6)	−0.0006 (6)	0.0005 (6)

### Geometric parameters (Å, º)

**Table:**

O1—C1	1.252 (2)	C10—H10B	0.9900
O2—C1	1.251 (2)	C11—C13	1.497 (4)
C1—C2'	1.547 (7)	C11—C12	1.520 (3)
C1—C2	1.558 (4)	C11—H11	1.0000
C2—C3	1.522 (4)	C12—H12A	0.9800
C2—C4	1.546 (4)	C12—H12B	0.9800
C2—H2	0.983 (17)	C12—H12C	0.9800
C3—H3A	0.9800	C13—H13A	0.9800
C3—H3B	0.9800	C13—H13B	0.9800
C3—H3C	0.9800	C13—H13C	0.9800
C2'—C3'	1.518 (11)	O3—C14	1.414 (2)
C2'—C4	1.548 (8)	O3—H3	0.90 (3)
C2'—H2'	0.98 (2)	O4—C15	1.422 (2)
C3'—H3'1	0.9800	O4—H4	0.79 (2)
C3'—H3'2	0.9800	O5—C16	1.4174 (19)
C3'—H3'3	0.9800	O5—H5A	0.86 (2)
C4—C5	1.369 (3)	N1—C17	1.495 (2)
C4—C7	1.383 (3)	N1—H1A	0.94 (2)
C5—C6	1.377 (3)	N1—H1B	0.94 (2)
C5—H5	0.9500	N1—H1C	0.91 (2)
C6—C9	1.381 (3)	C14—C17	1.526 (2)
C6—H6	0.9500	C14—H14A	0.9900
C7—C8	1.387 (3)	C14—H14B	0.9900
C7—H7	0.9500	C15—C17	1.524 (2)
C8—C9	1.380 (3)	C15—H15A	0.9900
C8—H8	0.9500	C15—H15B	0.9900
C9—C10	1.509 (3)	C16—C17	1.526 (2)
C10—C11	1.513 (4)	C16—H16A	0.9900
C10—H10A	0.9900	C16—H16B	0.9900
			
O2—C1—O1	123.26 (16)	C11—C10—H10B	108.6
O2—C1—C2'	109.6 (3)	H10A—C10—H10B	107.6
O1—C1—C2'	124.5 (3)	C13—C11—C10	114.0 (2)
O2—C1—C2	122.54 (17)	C13—C11—C12	110.4 (2)
O1—C1—C2	113.51 (18)	C10—C11—C12	110.5 (2)
C3—C2—C4	112.7 (3)	C13—C11—H11	107.2
C3—C2—C1	112.0 (3)	C10—C11—H11	107.2
C4—C2—C1	107.6 (2)	C12—C11—H11	107.2
C3—C2—H2	107.2 (18)	C11—C12—H12A	109.5
C4—C2—H2	111.5 (17)	C11—C12—H12B	109.5
C1—C2—H2	105.7 (18)	H12A—C12—H12B	109.5
C2—C3—H3A	109.5	C11—C12—H12C	109.5
C2—C3—H3B	109.5	H12A—C12—H12C	109.5
H3A—C3—H3B	109.5	H12B—C12—H12C	109.5
C2—C3—H3C	109.5	C11—C13—H13A	109.5
H3A—C3—H3C	109.5	C11—C13—H13B	109.5
H3B—C3—H3C	109.5	H13A—C13—H13B	109.5
C3'—C2'—C1	101.9 (6)	C11—C13—H13C	109.5
C3'—C2'—C4	103.9 (7)	H13A—C13—H13C	109.5
C1—C2'—C4	108.0 (4)	H13B—C13—H13C	109.5
C3'—C2'—H2'	97 (3)	C14—O3—H3	108.7 (12)
C1—C2'—H2'	114 (4)	C15—O4—H4	109.7 (13)
C4—C2'—H2'	127.3 (18)	C16—O5—H5A	109.7 (11)
C2'—C3'—H3'1	109.5	C17—N1—H1A	114.1 (12)
C2'—C3'—H3'2	109.5	C17—N1—H1B	110.8 (12)
H3'1—C3'—H3'2	109.5	H1A—N1—H1B	105.3 (16)
C2'—C3'—H3'3	109.5	C17—N1—H1C	110.3 (13)
H3'1—C3'—H3'3	109.5	H1A—N1—H1C	104.9 (17)
H3'2—C3'—H3'3	109.5	H1B—N1—H1C	111.3 (17)
C5—C4—C7	117.28 (18)	O3—C14—C17	109.37 (14)
C5—C4—C2	114.7 (2)	O3—C14—H14A	109.8
C7—C4—C2	128.0 (2)	C17—C14—H14A	109.8
C5—C4—C2'	141.6 (3)	O3—C14—H14B	109.8
C7—C4—C2'	100.3 (3)	C17—C14—H14B	109.8
C4—C5—C6	121.6 (2)	H14A—C14—H14B	108.2
C4—C5—H5	119.2	O4—C15—C17	111.56 (13)
C6—C5—H5	119.2	O4—C15—H15A	109.3
C5—C6—C9	121.9 (2)	C17—C15—H15A	109.3
C5—C6—H6	119.1	O4—C15—H15B	109.3
C9—C6—H6	119.1	C17—C15—H15B	109.3
C4—C7—C8	121.1 (2)	H15A—C15—H15B	108.0
C4—C7—H7	119.5	O5—C16—C17	112.09 (12)
C8—C7—H7	119.5	O5—C16—H16A	109.2
C9—C8—C7	121.6 (2)	C17—C16—H16A	109.2
C9—C8—H8	119.2	O5—C16—H16B	109.2
C7—C8—H8	119.2	C17—C16—H16B	109.2
C8—C9—C6	116.57 (19)	H16A—C16—H16B	107.9
C8—C9—C10	122.1 (2)	N1—C17—C15	107.22 (13)
C6—C9—C10	121.3 (2)	N1—C17—C14	107.80 (12)
C9—C10—C11	114.6 (2)	C15—C17—C14	110.87 (13)
C9—C10—H10A	108.6	N1—C17—C16	109.01 (13)
C11—C10—H10A	108.6	C15—C17—C16	110.98 (12)
C9—C10—H10B	108.6	C14—C17—C16	110.83 (14)
			
O2—C1—C2—C3	14.9 (4)	C2—C4—C7—C8	177.6 (2)
O1—C1—C2—C3	−174.3 (3)	C2'—C4—C7—C8	171.4 (3)
O2—C1—C2—C4	139.3 (2)	C4—C7—C8—C9	1.2 (4)
O1—C1—C2—C4	−50.0 (3)	C7—C8—C9—C6	−0.3 (3)
O2—C1—C2'—C3'	−83.9 (6)	C7—C8—C9—C10	179.1 (2)
O1—C1—C2'—C3'	114.1 (6)	C5—C6—C9—C8	−1.4 (3)
O2—C1—C2'—C4	167.0 (4)	C5—C6—C9—C10	179.2 (2)
O1—C1—C2'—C4	5.0 (7)	C8—C9—C10—C11	−90.4 (3)
C3—C2—C4—C5	−140.2 (3)	C6—C9—C10—C11	89.0 (3)
C1—C2—C4—C5	95.8 (3)	C9—C10—C11—C13	65.5 (3)
C3—C2—C4—C7	41.7 (4)	C9—C10—C11—C12	−169.5 (3)
C1—C2—C4—C7	−82.2 (3)	O4—C15—C17—N1	−55.72 (17)
C3'—C2'—C4—C5	−59.3 (7)	O4—C15—C17—C14	−173.15 (14)
C1—C2'—C4—C5	48.5 (8)	O4—C15—C17—C16	63.24 (18)
C3'—C2'—C4—C7	132.5 (5)	O3—C14—C17—N1	−58.01 (17)
C1—C2'—C4—C7	−119.7 (4)	O3—C14—C17—C15	59.06 (18)
C7—C4—C5—C6	−1.3 (3)	O3—C14—C17—C16	−177.23 (13)
C2—C4—C5—C6	−179.6 (2)	O5—C16—C17—N1	−56.68 (17)
C2'—C4—C5—C6	−168.2 (5)	O5—C16—C17—C15	−174.56 (13)
C4—C5—C6—C9	2.3 (3)	O5—C16—C17—C14	61.80 (18)
C5—C4—C7—C8	−0.4 (3)		

### Hydrogen-bond geometry (Å, º)

**Table:**

*D*—H···*A*	*D*—H	H···*A*	*D*···*A*	*D*—H···*A*
O3—H3···O2^i^	0.90 (3)	1.84 (3)	2.725 (2)	167 (2)
O4—H4···O1^ii^	0.79 (2)	1.92 (3)	2.689 (2)	163.7 (18)
O5—H5*A*···O1	0.86 (2)	2.57 (2)	3.0825 (19)	119.5 (17)
O5—H5*A*···O2	0.86 (2)	1.88 (2)	2.730 (2)	168.1 (18)
N1—H1*A*···O1	0.94 (2)	1.85 (2)	2.763 (2)	162.9 (18)
N1—H1*B*···O4^iii^	0.94 (2)	2.09 (2)	2.9224 (19)	146.7 (16)
N1—H1*C*···O5^iv^	0.91 (2)	1.97 (2)	2.806 (2)	152.1 (18)

Symmetry codes: (i) −*x*, *y*+1/2, −*z*+3/2; (ii) −*x*, *y*−1/2, −*z*+3/2; (iii) −*x*, −*y*+1, −*z*+2; (iv) *x*, −*y*+3/2, *z*+1/2.
